# Robotische Hernienchirurgie III: Robotische Inzisionalhernienversorgung mit „transversus abdominis release“ (r‑TAR). Videobeitrag und Ergebnisse einer Kohortenstudie

**DOI:** 10.1007/s00104-021-01480-z

**Published:** 2021-08-18

**Authors:** Ulrich A. Dietz, O. Yusef Kudsi, Miguel Garcia-Ureña, Johannes Baur, Michaela Ramser, Sladjana Maksimovic, Nicola Keller, Jörg Dörfer, Lukas Eisner, Armin Wiegering

**Affiliations:** 1 Klinik für Viszeral‑, Gefäss- und Thoraxchirurgie, Kantonsspital Olten, Baslerstrasse 150, 4600 Olten, Schweiz; 2 Department of Surgery, Good Samaritan Medical Center, 235 North Pearl St., 02301 Brockton, MA USA; 3 Hospital Universitario del Henares, Universidade Francisco de Vitoria, 28223 Pozuelo de Alarcón, Madrid Spanien; 4 Klinik für Allgemein‑, Viszeral- und Gefässchirurgie, Kantonsspital Baden, Im Engel 1, 5404 Baden, Schweiz; 5 Klinik und Poliklinik für Allgemein‑, Viszeral‑, Transplantations‑, Gefäß- und Kinderchirurgie, Universitätsklinikum Würzburg, Oberdürrbacher Straße 6, 97080 Würzburg, Deutschland

**Keywords:** Robotik, Inzisionale Hernie, Ventrale Hernie, Retromuskuläres Netz, Posteriore Komponentenseparation, Robotic surgical procedures, Incisional hernia, Ventral hernia, Retromuscular mesh, Posterior component separation

## Abstract

Das Prinzip der gezielten Trennung bzw. Schwächung einzelner Komponenten der Bauchdecke zur Spannungsentlastung der Medianlinie bei großen abdominellen Rekonstruktionen ist seit über 30 Jahren als anteriore Komponentenseparation (aKS) bekannt und ein etabliertes Verfahren. Auf der Suche nach Alternativen mit geringerer Komplikationsrate wurde die posteriore Komponentenseparation (pKS) entwickelt; der „transversus abdominis release“ (TAR) ist eine nervenschonende Modifikation der pKS. Mit den ergonomischen Ressourcen der Robotik (z. B. abgewinkelte Instrumente) kann der TAR minimal-invasiv durchgeführt werden (r-TAR): Bruchlücken von bis zu 14 cm lassen sich verschließen und ein großes extraperitoneales Netz implantieren. In diesem Videobeitrag wird die Versorgung großer Inzisionalhernien in der r‑TAR-Technik präsentiert. Exemplarisch werden die Ergebnisse einer Kohortenstudie an 13 konsekutiven Patienten vorgestellt. Der Eingriff ist anspruchsvoll, die eigenen Ergebnisse sind – wie auch die Berichte aus der Literatur – ermutigend. Der r‑TAR entwickelt sich zur Königsdisziplin der Bauchdeckenrekonstruktion.

**Zusatzmaterial online:**

Die Onlineversion dieses Beitrags (10.1007/s00104-021-01480-z) enthält ein Video und eine intraoperative Checkliste.

## Abstract (en)

The principle of targeted separation or weakening of individual components of the abdominal wall to relieve tension in the median line during major abdominal reconstruction has been known for over 30 years as anterior component separation (aKS) and is an established procedure. In search of alternatives with lower complication rates, posterior component separation (pKS) was developed; transversus abdominis release (TAR) is a nerve-sparing modification of pKS. With the ergonomic resources of robotics (e.g., angled instruments), TAR can be performed in a minimally invasive manner (r-TAR): hernia gaps of up to 14 cm can be closed and a large extraperitoneal mesh implanted. In this video article, the treatment of large incisional hernias using the r‑TAR technique is presented. Exemplary results of a cohort study in 13 consecutive patients are presented. The procedure is challenging, but our own results—as well as reports from the literature—are encouraging. The r‑TAR is becoming the pinnacle procedure for abdominal wall reconstruction.

## Hintergrund

Die Rekonstruktion großer Inzisionalhernien bleibt auch nach all den Fortschritten der vergangenen Jahrzehnte eine Herausforderung: Risikoprofil der Patient*innen, Hernienbefund und das technisch Machbare konvergieren in der Hand des Experten zu einem für den Patienten annehmbaren Behandlungsplan, doch es gibt viele Grauzonen und sehr filigrane Einzelheiten zu berücksichtigen. Auch wenn konventionell laparoskopische Verfahren das Komplikationsrisiko solcher Eingriffe deutlich reduzieren, bleiben doch die intraperitoneale Netzlage und eine etwas höhere Rezidivrate als nicht gelöste Probleme [1].

Das Thema der Inzisionalhernienversorgung zum wiederholten Mal aufzuarbeiten, wäre ohne größeren Nutzen, wenn die Herausforderungen ihrer chirurgischen Therapie nicht so aktuell wären, wenn nicht immer wieder innovative Therapieverfahren das Operationsspektrum erweitern würden und wenn nicht ständig neue Erkenntnisse im Bereich der Individualtherapie gewonnen würden. Die Robotik – als hochpräzise Form der Laparoskopie – nimmt einen immer wichtigeren Stellenwert in der Hernienchirurgie ein. Dieser Videobeitrag ist der dritte Teil einer Serie zur robotischen Hernienchirurgie und behandelt den robotischen „transversus abdominis release“ (r-TAR). Teil I und Teil II behandeln die Leistenhernienversorgung (robotische transabdominelle präperitoneale Patchplastik, r‑TAPP; [2]) und die primär ventrale und inzisionale Hernienversorgung (robotische ventrale transabdominelle präperitoneale Patchplastik [rv-TAPP] und r‑Rives/robotische transabdominelle retromuskuläre umbilikale Patchplastik [TARUP]; [3]).

## Indikationen und Kontraindikationen

Die Indikationen zur endoskopisch-robotischen Reparation großer inzisionaler Hernien sind prinzipiell ähnlich wie die zu konventionellen laparoskopischen Verfahren und richten sich auch nach dem Risikoprofil des Patienten [4, 5]. Inzisionale Hernien mit einem Durchmesser von 8–14 cm sind eine geeignete Indikation für den robotischen Eingriff. Bei kleineren Hernien sollte alternativ an den robotischen Rives (r-Rives, bei Inzisionalhernien) oder die robotische ventrale TAPP (rv-TAPP, bei primär ventralen Hernien) gedacht werden [3]. Die Länge der Hernie ist für die Verfahrenswahl nicht so bedeutend, da die Präparation beim r‑TAR umfassend von subxiphoidal bis zum Spatium Retzii reicht.

Relative Kontraindikationen sind sehr schlanke Patienten, kombinierte mediane und laterale Bruchlücken sowie Status nach offenem Abdomen mit Haut-Mesh-Deckung des Darmkonvolutes (Syn.: Thiersch-Plastik, nach Karl Th. Thiersch, 1886).

## Patientenaufklärung

Das minimal-invasive Vorgehen und die Anwendung des Operationsroboters wird dargestellt. Es wird im Allgemeinen auf postoperative Komplikationen wie postlaparoskopische Schulterschmerzen, Nachblutung, Serombildung und das Auftreten chronischer Schmerzen oder Taubheitsgefühl der Haut hingewiesen. Bei schlankem Körperbau kann es im Bereich der Haut über der Hernienreparation zu einer Wulstbildung kommen, welche sich mit großer Wahrscheinlichkeit im Laufe der ersten 3 bis 6 Monate postoperativ vollständig glättet.

Die Punktionsstelle der Veres-Nadel links subkostal und die Rasur des Abdomens und des rechten Oberschenkels (für die Neutralelektrode) werden angesprochen. Als zu erwartende Rezidivrate werden die verfügbaren Ergebnisse der konventionellen Reparationen genannt (ca. 2–8 % auf 5 Jahre). Die Implantation eines nichtresorbierbaren, flachen, großporigen Netzes wird besprochen. Die Patienten werden über kosmetische Optimierungsmöglichkeiten der postoperativen Narbenbehandlung beraten.

Zur Anwendung des Roboters erklären wir den Patienten, dass es kein eigentlicher Roboter ist, sondern ein Präzisionsinstrument, das ausschließlich von Chirurg*innen geführt wird.

## Anästhesie und Lagerung

Am Tag der Operation, auf der Aufnahmestation, erfolgt ein letztes Gespräch mit dem Patienten, die Bruchlücke wird mit Filzstift auf die Haut markiert und die schriftliche Einwilligung kontrolliert. Der Zugang zur Bauchdecke erfolgt von beiden Patientenseiten, der DaVinci Xi (Intuitive Surgical, CA, USA) wird von den Füßen des Patienten angesteuert. Der Patient wird in Rückenlage auf den Trumpf-Operationstisch positioniert (Trumpf-Medical, Saalfeld, Deutschland), die Art der Lagerung der Arme ist für diesen Eingriff nicht relevant. Gesicht und Beatmungstubus werden mit einem am Operationstisch montierten Metallrahmen geschützt. Der Eingriff wird unter Vollnarkose durchgeführt; die Relaxation muss bis zum Ende des Eingriffes bzw. bis zum Abdocken des Robotersystems optimal sein, bei Bedarf wird die neuromuskuläre Blockade am Ende des Eingriffes antagonisiert. Die Patienten bekommen eine perioperative Antibiotikaprophylaxe mit Cefuroxim 1,5 g (alternativ Clindamycin 600 mg).

## Übersicht der relevanten Anatomie des „transversus abdominis release“

**Figure 1:**
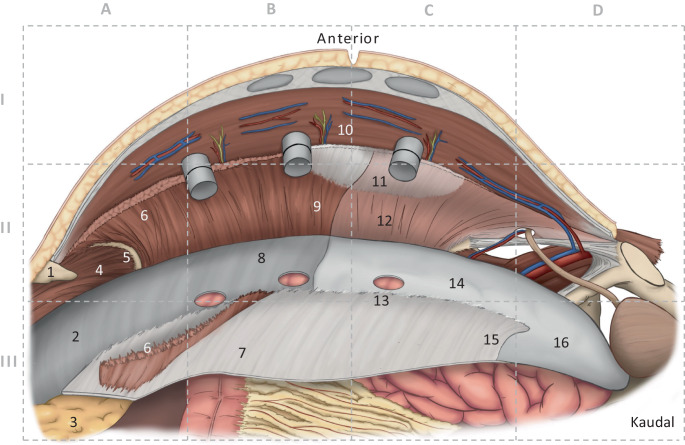

Die anterolaterale Bauchdecke wird von vier paarigen Muskeln gebildet, welche in der Mittellinie über die Linea alba mit den Muskeln der Gegenseite verbunden sind und eine funktionelle Einheit bilden. Von lateral/lumbal kommend enden die drei lateralen Muskeln (M. transversus abdominis, M. obliquus internus abdominis und M. obliquus externus abdominis) an der ipsilateralen Rektusscheide. Das mediale Ende der Muskelfasern des M. transversus abdominis bildet die Linea semilunaris (Spieghel-Linie). Muskuläre Fasern des *M. transversus abdominis* inserieren im kranialen Anteil fächerförmig an der Vorderseite der hinteren Rektusscheide (Abb. 1/6). Die *Interkostalnerven* erreichen den M. rectus abdominis von lateral kommend in der Schicht zwischen M. transversus abdominis und M. obliquus internus abdominis (Abb. 1/10 und 2a, b). Am *Rippenbogenrand* inserieren kranial das Zwerchfell, medial (im sternokostalen Winkel) der M. transversus abdominis und kaudal der M. obliquus internus abdominis. Der bei der r‑TAR-Präparation durchschimmernde Rippenbogenrand ist Leitstruktur und wird wegen der Eindrücklichkeit der Faserverläufe auch als „Wasserscheide“ beschrieben (Abb. 1/5). Die hintere Rektusscheide endet kaudal im Bereich der Linea arcuata (Douglas-Linie) (Abb. 1/15). Kaudal der Linea arcuata wird der M. rectus abdominis ausschließlich von seiner Faszie (Fascia recti propria, welche ein Teil der Fascia endoabdominalis ist) und vom Peritoneum bedeckt.

Insgesamt drei Leitstrukturen sind für die Absetzung der medialen Insertion des M. transversus abdominis von Bedeutung („transversus abdominis release“):

- kranial die Insertion des M. transversus abdominis auf der Vorderseite der hinteren Rektusscheide (Abb. 1/6),
- im mittleren Abschnitt die laterale Grenze der hinteren Rektusscheide und
- kaudal das Ende des Bogros-Raums an der Linea arcuata (Abb. 1/15).

Filip Muysoms bemerkt zu Recht, dass die etablierte TAR-Terminologie nicht ganz korrekt ist und es sich de facto um einen „posterior rectus sheath release“ handelt.

**Figure 2:**
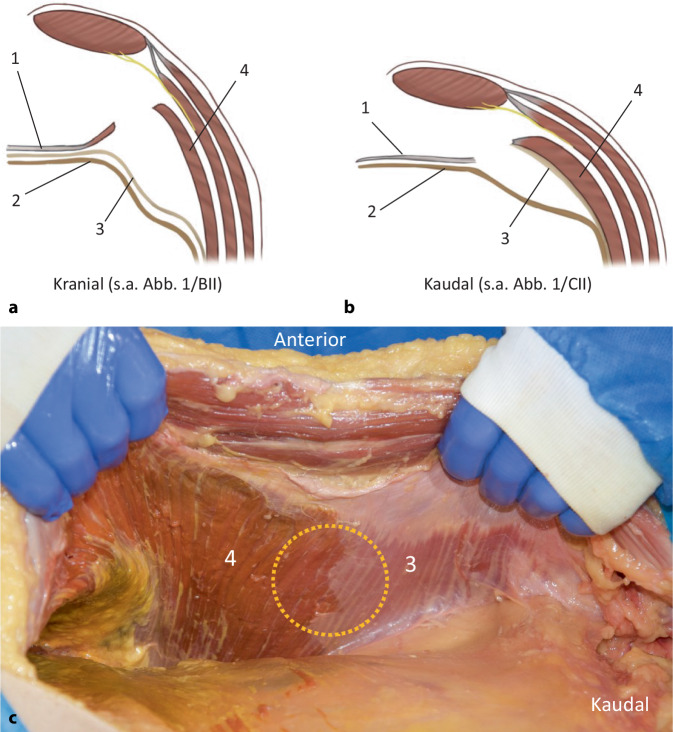

Das *Peritoneum parietale* hat 6 Schichten:

- das Mesothel,
- die Basalmembran
- die oberflächliche Wellenkollagenschicht
- das oberflächliche elastische Netz,
- das tiefe elastische Netz und
- die tiefe kollagenelastische Schicht.

Im Bereich der hinteren Bauchwand und des Zwerchfells hat das Peritoneum ein reiches Lymphkapillarnetz mit einer oberflächlichen und einer tiefen Schicht [6]. Das Peritoneum hat lymphatische Stomata (erstmals 1863 von Friedrich Daniel von Recklinghausen beschrieben, der auch in Würzburg tätig war), welche die Peritonealhöhle mit dem submesothelialen Lymphkapillarnetz verbindet; diese *peritonealen lymphatischen Stomata* kommen überwiegend am Peritoneum des Zwerchfells, des Ligamentum falciforme, der Ovarien und des Beckens vor [7]. Aus ultrastruktureller Sicht sind bei Mesothelzellen die apikalen Mikrovilli der Oberfläche und die interzellulären Junktionen von Bedeutung; Stomata befinden sich oft in Nähe der „milky spots“ und entstehen an der Angrenzung von drei Mesothelzellen; diese Stomata sind die wichtigsten Strukturen für die Drainage der peritonealen Flüssigkeit (unter physiologischen Bedingungen 5–100 ml); der negative intrathorakale Druck und die Zwerchfellbewegungen beeinflussen den intraperitonealen hydrostatischen Druck und bewegen die Flüssigkeit nach oben [8]. Das Mesothel regeneriert sehr schnell durch Metaplasie der subperitonealen Fibroblasten [9].

Bei der Präparation der hinteren Rektusscheiden und Ablösungen derselben vom Xiphoid entsteht das von Joachim Conze beschriebene „fatty triangle“ [10]. Die korrekte Präparation des „fatty triangle“ erlaubt es, bei der Retrorektusreparation (sowohl bei offenen Verfahren als auch beim r‑TAR) das Netz hinter dem Xiphoid zu positionieren und so eine sichere Unterfütterung des Netzes im kranialen Narbenhernienpol zu gewährleisten (Abb. 1/3). Kranial des „fatty triangle“ ist das Centrum tendineum des Zwerchfells. Indem das breitflächig mit präperitonealem Fettgewebe ausgefüllte Ligamentum falciforme an der vorderen Bauchdecke hängt und die Hinterseite der Linea alba unterfüttert, bildet es bei Ablösung der hinteren Rektusscheiden vom Xiphoid eine durchgehende verbindende Schicht zwischen rechter und linker hinterer Rektusscheide ([4, 10]; Abb. 1/3). Die kaudale Präparation der hinteren Rektusscheide endet an der Linea arcuata (Abb. 1/15), hier beginnt der präperitoneale Raum der retrosymphysär im *Spatium Retzii* (Abb. 1/16) und lateral zur Fascia iliaca hin im *Bogros-Raum* endet. Die laterodorsale Ablösung des Peritoneums und der Fascia endoabdominalis vom M. transversus abdominis reicht nach lumbal hinter der Gerota-Faszie zum M. quadratus lumborum.

Nach der aktuellen International Classification of Abdominal Wall Planes (ICAP), wird beim r‑TAR die Ebene „H“ („retromuscular plane“) präpariert, welche anterior vom M. rectus abdominis und dem M. transversus abdominis sowie posterior von der hinteren Rektusscheide (medial) und der Fascia transversalis (lateral) bzw. unterhalb der Linea arcuata nur von der Fascia transversalis gebildet wird [11]. Wir vermissen in der ICAP-Definition eine Unterscheidung zwischen der Fascia endoabdominalis und der Fascia transversalis, welche gerade auch in Abgrenzung zu der bei medialen Leistenhernien geschwächten Fascia transversalis zu terminologischer Verwirrung führen kann. Hier scheint auch eine Diskrepanz zwischen dem Terminologiekonsens des ICAP und den Präparationsbefunden beim r‑TAR evident: Sowohl an Anatomiepräparaten (z. B. in Abb. 2c) als auch bei sämtlichen r‑TAR-Operationen beinhaltet das hintere Präparationsblatt der r‑TAR nur im kranialen Anteil die Faszie des M. transversus abdominis (von Parker et al. als Fascia transversalis benannt), während im kaudalen Bereich diese fest am M. transversus abdominis haftet (Abb. 1/12 und 2/3; [12]). Möglicherweise erfolgte der terminologische Konsens etwas verfrüht und die Stimmen von Hernienexperten ohne klinische bzw. anatomische Erfahrung mit der TAR-Ebene wurden mit gewichtet. Hierzu sind weitere Studien notwendig. Im kranialen Bereich, wo die Faszie des M. transversus breitflächig von den Muskelfasern abgelöst wird (Abb. 1/9 und 2/4), kann es zu kleinsten flächigen Blutungen aus dem Kapillarnetz des *Epimysiums* und des *Perimysiums* kommen.

## Robotischer „transversus abdominis release“

Das WHO-Team-Time-out ist obligatorisch, gefolgt vom Repetieren der Operationsschritte auf der intraoperativen Checkliste (Supplement Material 1). Das Pneumoperitoneum wird über Veres-Nadel links-subkostal (12 mm Hg) angelegt. Die ersten 3 von insgesamt 6 Ports werden (nach Ropivacain-Infiltration) zunächst links-lateral positioniert. Wir arbeiten am DaVinci Xi mit 4 bezogenen Armen. Für die linke Seite werden die Arme #2 (8 mm), #3 (8 mm, Optik) und #4 (12 mm), für die rechte Seite die Arme #1 (8 mm), #2 (8 mm, Optik) und #3 (8 mm) verwendet. Als Instrumente verwenden wir eine 30°-Optik, die monopolare Schere (Hot Shears MCS; mit der wir die gesamte Blutstillung machen), den Prograsp Forceps und den Nadelhalter (Mega SutureCut Needle Driver). Alternativ kann mit einer bipolaren Fasszange (Fenestrated Bipolar Forceps oder Maryland Bipolar Forceps) präpariert und koaguliert werden. Zunächst arbeiten wir über die Ports auf der linken Patientenseite, der DaVinci Xi steht zu den Füßen des Patienten, wir verzichten auf die Systemzielausrichtung („Targeting“) und gestalten die Ausrichtung manuell (der DaVinci Xi hat kein spezifisches Targeting-Programm für den Zugang von den Füßen aus). Der Eingriff beginnt mit der explorativen Laparoskopie, je nach Befund erfolgt zunächst die komplette Adhäsiolyse der vorderen Bauchdecke.

### 1. Schritt (Zusatzmaterial online Videosequenz 00:44 min).

Begonnen wird über die linksseitigen Trokare mit der Präparation auf der rechten Seite. Die hintere Rektusscheide wird am medialen Rand (dem eigentlichen Bruchrand) vom Xiphoid bis zum Spatium Retzii eröffnet. Die hintere Rektusscheide wird bis zu ihrer lateralen Begrenzung freigelegt, Sorgfalt gilt der Schonung der epigastrischen Gefäße und der Interkostalnerven, die von lateral kommend in den M. rectus abdominis führen.

### 2. Schritt (Zusatzmaterial online Videosequenz 02:14 min).

**Figure 3:**
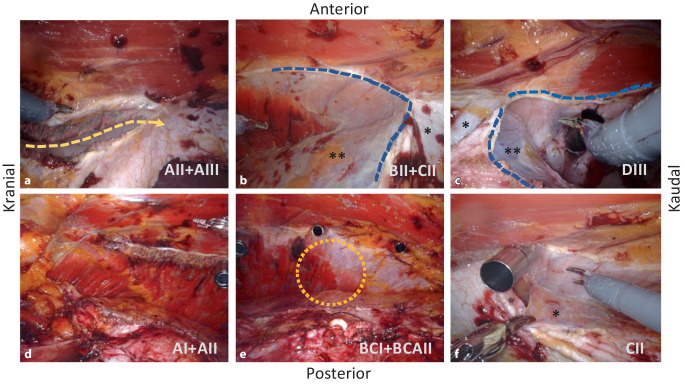

Die laterale Ablösung der posterioren Rektusscheide kann sowohl kranial („top-down“) oder kaudal („down-to-up“) beginnen. Beim Top-down-Beginn (Abb. 3a) werden die Fasern des M. transversus abdominis, die an der hinteren Rektusscheide inserieren, durchtrennt und die Fascia endoabdominalis (bzw. Faszie des M. transversus) nach lateral abgelöst; von da aus wird die Präparation in beide Richtungen weitergeführt, nach kranial über den Rippenbogenrand weit auf das Zwerchfell (Abb. 3d), und nach kaudal (Abb. 3b) in den Bogros-Raum und das Spatium Retzii. Die Insertion der hinteren Rektusscheide wird auch vom Xiphoid abgetrennt, damit entsteht ein weiter Zugang zum Zwerchfell und zu der Präparation des „fatty triangle“. Vorsicht gilt in diesem Bereich atypisch verlaufenden muskulären Fasern des Zwerchfells, die am Rippenbogenrand mit dem M. transversus abdominis verwechselt werden können (Abb. 3d). Beim „down-to-up“ erfolgt der Einstieg im Bereich der Linea arcuata (Abb. 3c) und wird in kranialer Richtung bis zur Durchtrennung der Muskelfasern des M. transversus abdominis und der Freilegung des „fatty triangle“ und des Zwerchfells fortgeführt. Auffallend ist, dass die Faszie des M. transversus abdominis im kranialen Anteil der Präparation am Peritoneum bleibt (Abb. 1/9 und 2/4) und im kaudalen Bereich am M. transversus abdominis haftet (Abb. 1/12, 2/3 und 3e). Nach lateral wird die Präparation weit nach lumbal fortgeführt, bis die nun abgelöste innere Bauchwandschicht locker auf den Darmschlingen liegt. Mit dem Prograsp Forceps wird diese nun probatorisch nach medial gezogen, um die Spannungsfreiheit für den späteren Nahtverschluss zu überprüfen. Die kontralateralen Ports werden unter Sicht eingeführt. Hiermit endet die erste Hälfte der Präparation und der DaVinci Xi wird abgekoppelt.

### 3. Schritt (Zusatzmaterial online Videosequenz 04:41 min).

**Figure 4:**
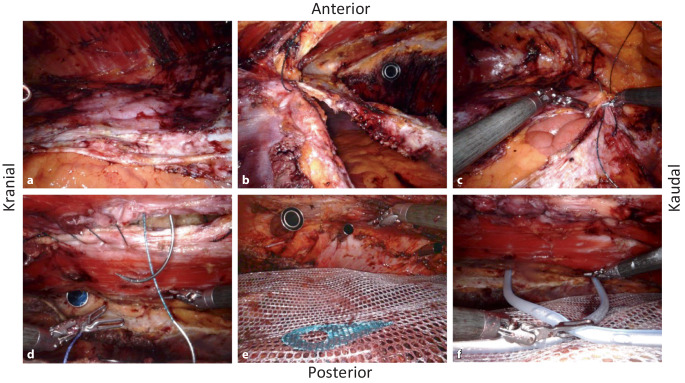

Wir drehen das Stativ der DaVinci-Xi-Arme manuell (Seitenknopf an den Armen #1 und #4) um 180° und docken 3 Arme manuell (ohne System-Targeting) an die Ports. Nun wird über die rechtsseitigen Ports analog die linksseitige Bauchdecke präpariert. Bei der lateralen Ablösung der hinteren Rektusscheide und der Fascia endoabdominalis sind nun die transabdominell positionierten ersten 3 Ports „im Wege“, diese müssen nacheinander extraperitonealisiert werden, damit die Präparation nach lateral fortgeführt werden kann. Die hierdurch entstandenen 3 Löcher des Peritoneums (Abb. 1/13 und 3f) werden mit resorbierbarem Faden vernäht. Mit dem Prograsp Forceps wird nun die linksseitige innere Bauchdeckenschicht mobilisiert und die Spannungsfreiheit für den anschließenden Nahtverschluss geprüft (Abb. 4a).

### 4. Schritt (Zusatzmaterial online Videosequenz 06:47 min).

Die spannungsfrei auf den Darmschlingen liegenden hinteren Rektusscheiden werden median mit einem 30 cm langen V‑Loc-180/0-USP-Faden mit GS-21-Nadel (Medtronic Deutschland) verschlossen; wir beginnen mit zwei Fäden, einer von kranial aus dem Bereich des „fatty triangle“ kommend (Abb. 4b) und einer kaudal, aus dem Spatium Retzii kommend (Abb. 4c). Der Pneumoperitoneumdruck wird über 3 min auf 4 mm Hg reduziert, um eventuelle Flächenblutungen aus dem M. transversus abdominis zu erkennen und die Hämostase zu vervollständigen. Nun wird entschieden, ob auch die anterioren Rektusscheiden (Linea alba) robotisch verschlossen werden oder ob der Eingriff eine offene Hautresektion (Hybrideingriff) braucht.

### 5. Schritt (Zusatzmaterial online Videosequenz 08:34 min).

Abschließend wird bei reduziertem Pneumopräperitoneumdruck (8 mm Hg) die mediane Linea alba von subxiphoidal und infraumbilikal kommend schrittweise mit V‑Loc-180/0-USP-Naht (mit GS-21-Nadel) gerafft (Abb. 4d), der Bruchsack wird zur Seromprophylaxe punktuell mitgefasst. Das Netz muss bei der effektiv weiten Überlappung nicht fixiert werden. Die Durchtrittstellen der Ports durch die muskuläre Bauchdecke werden vom Netz unterfüttert und müssen nicht transfaszial verschlossen werden. Alternativ wird bei geplantem Hybrideingriff anstatt Schritt 5 mit Schritt 6 fortgefahren.

### 6. Schritt (Zusatzmaterial online Videosequenz 10:45 min).

Über den 12-mm-Port wird das meist 30 × 30 cm messende Versatex-Netz (Polyester, monofilamentär, großporig; Medtronic Deutschland) eingeführt und so ausgebreitet, dass es im Bereich des „fatty triangle“ das Xiphoid unterfüttert und von da aus bis zur Symphyse reicht; nach lateral reicht das Netz beidseits bis in die Lendenregion (Abb. 4e).

### 7. Schritt (Zusatzmaterial online Videosequenz 11:42 min).

Additiv wird die große Präparationsfläche zur Sicherung der kapillaren Blutstillung mit Arista AH (resorbierbares Hämostyptikum aus pflanzlicher Stärke in Puderform; BD Deutschland) mittels FlexTip-XL-R-Applikator (BD Deutschland) besprüht. Über die linksseitigen Ports werden 2 Passivdrainagen eingebracht, eine nach subdiaphragmal (Drainage #1) und eine ins Spatium Retzii (Drainage #2; Abb. 4f). Es erfolgt die Zählkontrolle der Instrumente und Operationsmaterialien und die Entlastung des Pneumoperitoneums unter Sicht.

In der Hybridvariante (rh-TAR) werden Netzpositionierung, Arista-Applikation und Drainageneinlage wie oben beschrieben robotisch durchgeführt (Schritte 6 und 7), erst danach wird abgedockt und der Eingriff offen beendet: Hautnarbe und subkutaner Bruchsack werden reseziert, die Linea alba wird mit einer fortlaufenden Everett-Naht (nach William G. Everett, aus Cambridge, England, 1970; mit einem Nahtlängen-zu-Wundlängen-Verhältnis von 4:1) rekonstruiert und die Haut mit intrakutaner Naht verschlossen.

## Kasuistik und Studiendesign

Dieser Videobeitrag fasst die Erfahrungen der Operationen zusammen, welche von Juni 2019 bis Dezember 2020 durchgeführt wurden. Es handelt sich um eine Kohortenstudie ohne Kontrollgruppe. Die Studie wurde von der zuständigen Ethikkommission der Nordwestschweiz bewilligt (Ref. Nr. 2019-02046). Die Entscheidung, ob ein Eingriff ausschließlich robotisch oder im Hybridverfahren durchgeführt wurde, richtete sich nach den jeweiligen Befunden. Die Patienten wurden 6 Wochen postoperativ klinisch und bei Bedarf auch sonographisch nachkontrolliert. Die 1‑Jahres-Nachuntersuchungen sind in dieser Kohorte geplant, liegen zum Zeitpunkt der Publikation jedoch noch nicht vor. Sämtliche Daten wurden pseudonymisiert in einer klinikinternen Datenbank erfasst, die passwortgeschützt den Untersuchern zugänglich ist. Der *t*-Test wurde zum Vergleich der Menge der Drainagenflüssigkeit, Dauer der Belassung der Drainage und des stationären Aufenthalts verwendet. Ein *p*-Wert unter 0,05 wurde als signifikant gewertet.

## Ergebnisse

Es wurden 13 Patienten versorgt, das durchschnittliche Alter betrug 58,2 Jahre (Range: 38–74), 30,8 % waren Frauen, der durchschnittliche Body-Mass-Index (BMI) betrug 29,9 kg/m^2^ (Range: 24,8–37,2). Die häufigsten Nebenerkrankungen waren arterielle Hypertonie (76,9 %) und „chronic obstructive pulmonary disease“ (COPD; 30,8 %). Vier Patienten (30,8 %) waren oral antikoaguliert, 8 Patienten (61,5 %) hatten einen ASA(American Society of Anesthesiology)-Score II, 5 Patienten (38,5 %) einen ASA-Score III (Tab. 1).

**Table 1:** 

	r‑TAR/rh-TAR (*n* = 13)		
**Alter (MW, Range [SA])**	58,2	38–74	(±12,6)
**Geschlecht weiblich (*n* [%])**	4	–	(30,8)
**BMI kg/m**^**2**^** (MW, Range [SA])**	29,9	24,8–37,2	(±4,0)
**Raucher (*n* [%])**	7	–	(53,8)
**Ethnie (*n* [%])**			
*Nordeuropa*	11	–	(84,6)
*Mediterran*	2	–	(15,4)
**Art der beruflichen Tätigkeit (*n *[%])**			
*Schreibtisch*	3	–	(23,1)
*Körperlich anstrengend*	2	–	(15,4)
*Keine Arbeit oder berentet*	5	–	(38,5)
*Nicht angegeben*	3	–	(23,1)
**Komorbiditäten (*n* [%])**			
*Arterielle Hypertonie*	10	–	(76,9)
*Koronare Herzkrankheit*	1	–	(7,6)
*Diabetes mellitus*	3	–	(23,1)
*COPD*	4	–	(30,8)
*Thrombembolisches Ereignis in Anamnese*	–	–	–
*Immunsuppressive Therapie*	–	–	–
*Orale Antikoagulation*	4	–	(30,8)
DOAC	2	–	(15,4)
Marcumar	–	–	–
Plättchenaggregationshemmer	2	–	(15,4)
*ASA-Score*			
I	–	–	–
II	8	–	(61,5)
III	5	–	(38,5)
*CCI (MW [SA])*	9,3	–	(±12,7)

*ASA* American Society of Anesthesiology, *CCI* Charlson Komorbiditätsindex, *COPD* „chronic obstructive pulmonary disease“, *DOAC* duale orale Antikoagulation, *MW* Mittelwert, *Range* Variationsbreite, *r‑TAR/rh-TAR *robotischer „transversus abdominis release“/Hybridvariante, *SA* Standardabweichung

Sämtliche Hernien waren Inzisionalhernien, in einem Fall bestand die Kombination einer medianen Hernie mit einer paramedianen (nach Rückverlagerung eines Ileostomas), bei einem Patienten bestand eine ausschließlich paramediane Inzisionalhernie (8 × 8 cm, nach Rückverlegung eines Kolostomas). Häufigste Ursachen für die Inzisionalhernien waren die Operation eines kolorektalen Karzinoms (46,1 %) und Implantation einer Rohrprothese bei abdominellem Aortenaneurysma (15,4 %), bei 3 Patienten bestand ein Rezidiv (einmal nach laparoskopischem intraperitonealem Onlay-Mesh [IPOM], 2‑mal nach offener retromuskulärer Netzimplantation). Die Breite der Bruchlücken variierte von 7–16 cm, bei allen Patienten wurde die Linea alba morphologisch rekonstruiert. Die durchschnittliche Ratio von Netzgröße zu Bruchlückengröße betrug 8,2. Die Operationsdauer (Schnitt-Naht-Zeit) betrug im Durchschnitt 223,5 min (Range: 167–317 min), die Zeiten für das Andocken des DaVinci Xi, das intraoperative Umdocken sowie die Hybridhautresektion sind dabei mit inbegriffen. Vier Patienten (20,7 %) wurden im Hybridverfahren operiert. Alle weiteren Daten zu den Bruchlücken, der Netzgröße und den Drainagen sind in Tab. 2 dargestellt.

**Table 2:** 

	r‑TAR/rh-TAR (*n* = 13)		
		Range	
*Art der Hernie (n [%])*			
Umbilikal, epigastrisch oder Spieghel	–	–	–
Inzisional	13	–	(100,0)
*Vorerkrankung*			
Kolorektales Karzinom	6	–	(46,1)
Abdominelles Aortenaneurysma	2	–	(15,4)
Rezidiv einer primär ventralen Hernie	3	–	(23,0)
Andere, gutartig	3	–	(23,0)
*Größe der Bruchlücke*			
Länge in cm (MW, Range [SA])	14,9	8–24	(±4,9)
Breite in cm (MW, Range [SA])	11,1	7–16	(±3,0)
Defektfläche in cm^2^ (MW, Range [SA])	132,4	88–301	(±69,6)
*Bruchlückenverschluss (n [%])*	13	–	(100,0)
*Netzgröße*			
Länge in cm (MW, Range [SA])	31,7	29–45	(±4,7)
Breite in cm (MW, Range [SA])	28,8	25–30	(±2,1)
Netzfläche in cm^2^ (MW, Range [SA])	907,5	783–1125	(±80,2)
*Ratio Netzfläche:Bruchlückenfläche (MW, Range [SA])*	8,2	3,7–15,6	(±3,2)
*Netzart (n [%])*			
Versatex	13	–	(100,0)
*Netzfixation (n [%])*			
Keine	12	–	(99,3)
Vicrylnaht	1	–	(7,6)
*Drainageneinlage (n [%])*	10	–	(76,9)
*Hybridprozedur (rh-TAR)*	4	–	(20,7)
*Arista-Applikation*	8	–	(61,5)
*Schnitt-Naht-Zeit in min (MW, Range [SA])*^*a*^	223,5	167–317	(±43,5)

*MW* Mittelwert, *Range* Variationsbreite, *r‑TAR/rh-TAR* robotischer „transversus abdominis release“/Hybridvariante, *SA* Standardabweichung

^a^Die Zeit beinhaltet das Andocken, die Adhäsiolyse und das Umdocken

**Figure 5:**
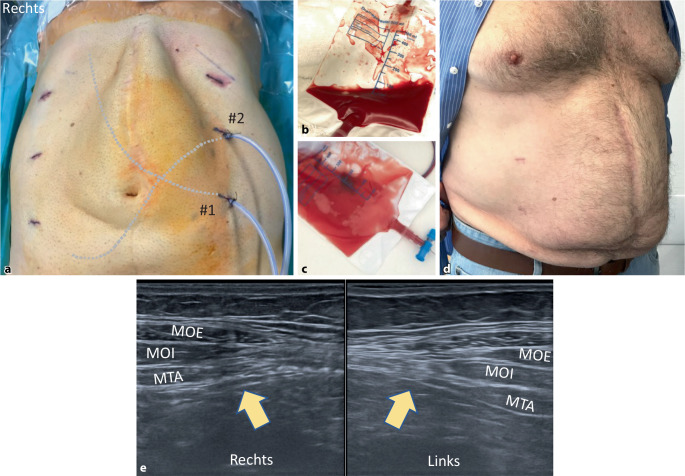

In Tab. 3 ist der postoperative Verlauf dargestellt. Der stationäre Aufenthalt betrug im Durchschnitt 4,7 Tage. Die subphrenischen Drainagen förderten im Durchschnitt mehr als die Drainagen im kleinen Becken (246 ml vs. 145 ml), allerdings ohne statistischen Unterschied (*p* = 0,181; Abb. 5b, c). Postoperativ traten bei 5 Patienten Wundereignisse auf: 2 Serome (15,3 %) und 3 Hämatome (23 %; [13]). Es ist weder eine Wundheilungsstörung noch eine Wundinfektion aufgetreten. Die Patienten wurden am Abend des Operationstages mobilisiert und bekamen ein leichtes Abendessen. Am 1. oder 2. postoperativen Tag hatten alle Patienten Stuhlgang. Zwei Patienten wurden wegen Nachblutung revidiert (Dindo-Clavien IIIb), der erste in offener, der zweite in robotischer Technik, bei keinem war bei der Hämatomausräumung eine chirurgische Blutungsquelle ersichtlich [14].

**Table 3:** 

	r‑TAR/rh-TAR (*n* = 13)			
		Range		*p* Wert
**Krankenhausaufenthaltsdauer in Tagen (MW [SA])**	4,7	–	(±2,9)	–
**VAS am 1. postoperativen Tag (MW [SA])**	3,7	–	(±2,4)	–
**Drainagenmenge (ml)**				
*Drainage #1 (subphrenisch) (MW, Range [SA])*	246,3	40–560	(±171,5)	*p* = 0,181
*Drainage #2 (Hypogastrium) (MW, Range [SA])*	145,6	20–410	(±150,1)	
**Dauer der Drainagen in situ (Tage)**				
*Drainage #1 (subphrenisch) (MW, Range [SA])*	3,1	1–5	(±1,2)	*p* = 0,292
*Drainage #2 (Hypogastrium) (MW, Range [SA])*	2,5	1–5	(±1,0)	
**Unerwünschte Ereignisse innerhalb 6 Wochen**				
*Wundereignisse (SSO) (n [%])*	5	–	(38,4)	–
Serom (*n* [%]) [13]	2	–	(15,3)	–
– Grad I	–	–	–	–
– Grad II	1	–	(7,6)	–
– Grad III	1	–	(7,6)	–
– Grad IV	–	–	–	–
Hämatom (*n* [%])	3	–	(23,0)	–
Wundinfektion (*n* [%])	–	–	–	–
*Ungeplante Wiedervorstellung bei Schmerzen (n [%])*	–	–	–	–
*Verzögertes Einsetzen der Darmpassage (n [%])*	–	–	–	–
*Thrombembolisches Ereignis (n [%])*	–	–	–	–
*Clavien-Dindo (n [%]) *[14]	4	–	(30,7)	–
Grad I	4	–	–	–
Grad II	–	–	–	–
Grad IIIa	1	–	–	–
Grad IIIb	2	–	–	–
Grad IV	–	–	–	–
**Nachuntersuchung nach 6 Wochen (*n* [%])**				
*Erfolgt*	13	–	(100,0)	–
*Rezidiv*	–	–	(0,0)	–
*Bauchdeckenschmerzen*	–	–	(0,0)	–
*Serom*	1	–	(7,6)	–
*Hämatom*	1	–	(7,6)	–

*MW* Mittelwert, *Range* Variationsbreite, *r‑TAR/rh-TAR *robotischer „transversus abdominis release“/Hybridvariante,* SA* Standardabweichung, *SSO* „surgical site occurrence“, *VAS* visuelle Analogskala für Schmerzeinschätzung

## Diskussion

Die Entwicklung des heutigen TAR begann 2008, als Alfredo Carbonell die posteriore Komponentenseparation (pKS) für die offene Inzisionalhernienversorgung beschrieben hat [15]. Bei der pKS wird lateral der hinteren Rektusscheide die Ebene zwischen M. obliquus internus und M. transversus abdominis präpariert (Ebene „E“ bzw. „retro-oblique plane“ bei ICAP; [11]). Allerdings besteht ein erhebliches Risiko der Läsion von Interkostalnerven, welche genau in dieser Ebene verlaufen. Bereits 4 Jahre später beschrieb Yuri Nowitzky den offenen TAR, eine technische Modifikation der pKS, welche die neurovaskulären Bündel schont und der Ebene „H“ bzw. dem „retromuscular plane“ bei ICAP entspricht [11, 16]. In einer Studie an Leichen, wurde der Medialisierungseffekt der verschiedenen beschriebenen Komponentenseparationen untersucht, die pKS bietet mit 9,4 cm je Seite eine messbar vorteilhafte Medialisierung im Vergleich zur anterioren Komponentenseparation, welche im Durchschnitt 5,8 cm erreicht [17]. Es kann aus anatomischen Überlegungen angenommen werden, dass der TAR, als Variante der pKS, mindestens vergleichbare Medialisierungsressourcen wie die pKS hat.

Nachdem in den vergangenen Jahren das laparoskopische IPOM sehr verbreitet war und vor allem für die geringe Wundkomplikation bekannt wurde, zeigt eine aktuelle Metaanalyse, dass Netze in Retrorektusposition die geringste Rezidivrate haben (Odds Ratio [OR] 0,281, 95 %-Konfidenzintervall[CI] 0,06–0,47) bei ebenfalls geringem Risiko an Netzinfektionen (OR 0,449, 95 % CI 0,12–1,16), wobei das IPOM (als „Underlay“ beschrieben) ein noch geringeres Risiko an Wundkomplikationen hat (OR 0,878, 95 % CI 0,29–1,99); im Vergleich zu Onlay, Inlay und Underlay (bzw. IPOM) wird in dieser Metaanalyse mit 94,2 % Wahrscheinlichkeit gefolgert, dass „retromuscular“ die beste Schicht für eine Netzimplantation ist [18]. Weitere Argumente gegen das IPOM sind, neben der erhöhten Rezidivrate und den postoperativen Schmerzen, die Komplikationen am Netz (Adhäsionen und Netzarrosionen), sodass heute die Extraperitonealisierung der Netze in Kombination mit den Vorteilen des minimal-invasiven Operierens im Vordergrund steht.

Der r‑TAR wurde erstmals 2017 von Warren et al. beschrieben [19]. In der Originalbeschreibung wird der Roboter von beiden Seiten angedockt und die laterale Präparation reicht bis zur Projektion der vorderen Axillarlinie; in dieser Serie werden die laparoskopische Technik (*n* = 103) mit der Robotik (*n* = 53) verglichen, wobei nur 43 % der robotischen Eingriffe als r‑TAR erfolgten [19]. In einer retrospektiven Studie zum Vergleich des offenen TAR (o-TAR; *n* = 76) und des r‑TAR (*n* = 26) war beim o‑TAR die Operationszeit zwar signifikant kürzer (287 vs. 365 min), die Morbidität aber beim o‑TAR höher (39 vs. 19 %, *p* = 0,09) und die Spitalaufenthaltszeit beim o‑TAR signifikant länger (6 vs. 3 Tage; [20]).

Eine Weiterentwicklung des r‑TAR ist der Hybrid-r-TAR (rh-TAR), bei dem die Hautnarbe und der Bruchsack am Ende der Operation reseziert werden und die Linea alba in offener Technik verschlossen wird. In einer Kohortenstudie an 20 rh-TAR-Operationen zeigten Kudsi et al., dass die Komplikationsrate gering und die Zufriedenheit der Patienten, gemessen am Qualitiy-of-life(QoL)-Score der Europäischen Herniengesellschaft (EHS; kosmetische Zufriedenheit und Behinderung bei der Arbeit), im Vergleich prä- zu postoperativ signifikant erhöht ist [21]. Die Operationsdauer der rh-TAR lag in der Serie von Kudsi bei 296,5 ± 94,5 min, die Bruchlückenfläche variierte von 204–333 cm^2^, die Netzfläche von 600–1050 cm^2^, die Ratio Netzfläche zu Bruchlückenfläche betrug im Durchschnitt 4,11; es kam zu 3 Seromen (15 %), 2 Wundinfektionen (10 %), bei 2 Patienten musste die Wunde revidiert werden (10 %), es trat bei einem Follow-up von durchschnittlich 319 Tagen kein Rezidiv auf [21]. In der Literatur wird der r‑TAR bei Bruchlücken mit einer Breite von 7–14 cm empfohlen, darunter (4–7 cm) ist der r‑Rives (retrorektales Netz) eine gute Option [3, 22].

In unserer Serie lag die Operationszeit mit 217 min im Mittelwert (Range 167–317 min) etwas unter dem Durchschnitt der Literatur; allerdings war auch die Bruchlückenfläche mit 132,4 cm^2^ (Range 88–301 cm^2^) etwas kleiner und der BMI mit 29,9 kg/m^2^ (Range 24,8–37,2 kg/m^2^) geringer als z. B. bei Kudsi et al. (Bruchlückenfläche 255 cm^2^ und BMI 33,5 kg/m^2;^ [21]). In der Anfangsphase haben wir zwei Revisionen wegen eines Hämatoms durchgeführt. Als Konsequenz haben wir unsere Operationsverfahren mit 3 Maßnahmen angepasst:

- Revision der Blutstillung nach Naht der hinteren Rektusscheiden unter reduziertem Pneumoperitoneum (4 mm Hg über 3 min),
- Applikation von Arista auf das Netz. um die kapillare Blutstillung auf der großflächigen Komponentenseparation zu unterstützen und
- Einlage von 2 Passivdrainagen (Robinson-Drainagen), subphrenisch und retropubisch.

Nach dieser Anpassung der Technik haben wir keinen Patienten mehr revidieren müssen.

Es scheint, dass gemessen an der Qualität und Quantität des Drainagensekrets die breitflächige Ablösung des Peritoneums vom Zwerchfell und der gesamten Bauchdecke zu einer passageren Disruption der physiologischen Resorptionsmechanismen der peritonealen Flüssigkeit z. B. durch die Unterbrechung der lymphatischen Wege und der peritonealen Stomata vor allem in der Topographie des Zwerchfells führt. Hier sind noch weitere Studien vonnöten. Ein großer Vorteil des r‑TAR ist, dass der Darm kaum berührt wird („no-touch“ der Darmserosa während des gesamten Eingriffes), was sich postoperativ durch die geringe Inzidenz an Ileus und die Möglichkeit des unmittelbaren Kostaufbaus zeigt. Einrisse des Peritoneums kommen sehr selten vor und können problemlos vernäht werden.

Es ist erstaunlich zu sehen, dass die weiten Bruchlücken (in unserer Serie von bis zu 16 cm Durchmesser) nach 2–3 h unter 12-mm-Hg-Pneumoperitoneum sowohl robotisch (r-TAR) als auch offen (rh-TAR) mit Naht adaptierbar sind; dies ist wahrscheinlich der Dehnung der Bauchdecke unter Muskelrelaxation unter gleichzeitigem Pneumoperitoneum zu verdanken, ein Effekt, der ähnlich zu sein scheint wie beim offenen AWEX(„abdominal wall expanding“)-System zum Verschluss der Bauchdecke nach Laparostoma [23]. Künftige Studien müssen erst noch die Dehnung der Bauchdecke unter Pneumoperitoneum während eines laparoskopischen Eingriffes definieren, um mittels Analyse der Morphologie der Bauchdeckenmuskulatur und Bruchlückengröße in der Computertomographie die Operationsplanung und Patientenberatung zu optimieren.

Auch wenn sich die ästhetische Zufriedenheit der Patienten beim r‑TAR nach 3 bis 6 Monaten mit der Glättung der Form der Bauchdecke ergibt (Abb. 5a, d), haben wir die gleiche Erfahrung wie Yusef Kudsi gemacht, dass die Zufriedenheit der Patienten nach dem rh-TAR unmittelbar nach der Operation hoch ist [21]. Bei postoperativen Ultraschallkontrollen nach 3 und 6 Monaten zeigt sich der M. transversus abdominis auf Ebene der Linea semilunaris unauffällig, ohne Zeichen von lateraler Retraktion oder Atrophie, die Morphologie und die Biomechanik der Bauchdecke bleiben nach dem r‑TAR erhalten (Abb. 5e).

Die Frage nach der optimalen Netzgröße muss in Zukunft weiter präzisiert werden. Die Grenze für die Netzgröße ist durch die Fläche der anterolateralen Bauchdecke (inklusive eines Teils des Zwerchfells, der Lendenregion und des Spatium Retzii) gegeben. Sicher ist, dass die Forderung einer Netzfläche-zu-Bruchfläche-Ratio von 16 nur für Bridging-Verfahren gilt und nicht bei Bruchlückenverschluss im Sinne der morphologischen und funktionellen Bauchdeckenrekonstruktion [24, 25]. Auch die Netzfixation an zahlreichen Punkten, wie für das Bridging Verfahren gefordert, scheint nicht auf großflächige extraperitonealisierte Netze mit Bruchlückenrekonstruktion übertragbar zu sein [26]. Eine unterstützende chemische Komponentenseparation mit Butolinumtoxin scheint bisher beim r‑TAR nicht nötig zu sein. Dass die Kosten des r‑TAR deutlich geringer sind als die des laparoskopischen IPOMs, sei nur am Rande erwähnt [3].

Der r‑TAR ist kein Eingriff für Beginner in der Robotik. Wir empfehlen, dass Interessierte an der Methode die Anatomie an der Leiche repetieren (z. B. im ORSI-Institut in Belgien) und zu Beginn Erfahrung mit dem robotischen Rives gewinnen [3]. Es ist auch sinnvoll, den ersten Eingriff mit Unterstützung eines Proctors zu machen, der sich in der Methode auskennt. Vor allem die Gefahr der Zwerchfellläsion, die Komplexität der Präparation des „fatty-triangle“, die Schonung der Interkostalnerven und die Navigationssicherheit im Bogros Raum sind – wie auch die stabile Naht der Linea alba – herausfordernd. Der r‑TAR ist (in Anlehnung an eine Formulierung von Patrick Süsskind, Das Parfüm, 1985) die genialste und innovativste Umsetzung all dessen, was wir in den letzten zwei Jahrzehnten über Bauchdeckenrekonstruktion gelernt haben, zwei Jahrzehnte die überaus reich an genialen und innovativen Ideen zur Bauchdeckenrekonstruktion waren. Der r‑TAR entwickelt sich zur Königsdisziplin der Hernienreparation, die minimal-invasive Extraperitonealisierung großer Netze ist wahrscheinlich sein größter Beitrag. Weitere Studien sind nötig, um die bisherigen Ergebnisse zu bestätigen oder zu falsifizieren.

## Fazit für die Praxis

- Das robotische Transversus-abdominis-release(r-TAR)-Verfahren vereint die Vorteile der offenen Reparation (morphologische und funktionelle Rekonstruktion) und der Laparoskopie (wenig Wundkomplikationen, geringer postoperativer Ileus, kurzer Spitalaufenthalt).
- Bruchlücken von 8–14 cm Breite können verschlossen werden.
- r‑TAR ermöglicht die Extraperitonealisierung großer Netze.
- Fortgeschrittene anatomische Kenntnisse sind erforderlich.
- Das Monitoring der Blutstillung unter niedrigem Druck ist wichtig, das postoperative Ablassen der serösen Flüssigkeit über Drainagen ist sinnvoll.
- Das Hybridverfahren (rh-TAR) ergibt zeitnah ein sehr gutes ästhetisches Ergebnis.

## Supplementary Information





