# Erratum zu: Robotische Hernienchirurgie III: Robotische Inzisionalhernienversorgung mit „transversus abdominis release“ (r-TAR). Videobeitrag und Ergebnisse einer Kohortenstudie

**DOI:** 10.1007/s00104-022-01573-3

**Published:** 2022-01-17

**Authors:** Ulrich A. Dietz, O. Yusef Kudsi, Miguel Garcia-Ureña, Johannes Baur, Michaela Ramser, Sladjana Maksimovic, Nicola Keller, Jörg Dörfer, Lukas Eisner, Armin Wiegering

**Affiliations:** 1 Klinik für Viszeral‑, Gefäss- und Thoraxchirurgie, Kantonsspital Olten, Baslerstrasse 150, 4600 Olten, Schweiz; 2 Department of Surgery, Good Samaritan Medical Center, 235 North Pearl St., 02301 Brockton, MA USA; 3 Hospital Universitario del Henares, Universidade Francisco de Vitoria, 28223 Pozuelo de Alarcón, Madrid Spanien; 4 Klinik für Allgemein‑, Viszeral- und Gefässchirurgie, Kantonsspital Baden, Im Engel 1, 5404 Baden, Schweiz; 5 Klinik und Poliklinik für Allgemein‑, Viszeral‑, Transplantations‑, Gefäß- und Kinderchirurgie, Universitätsklinikum Würzburg, Oberdürrbacher Straße 6, 97080 Würzburg, Deutschland

**Erratum zu:**

**Chirurg 2021**

10.1007/s00104-021-01480-z

Dieser Beitrag zum Thema „Robotische Hernienchirurgie. r‑TAR“ ist der dritte Teil einer Reihe von mehreren Beiträgen zum Thema. Titel und Untertitel wurden angepasst. Die Originalversion dieses Beitrags wurde korrigiert.

