# Supplementary material for: Robotic hernia repair III. English version: Robotic incisional hernia repair with transversus abdominis release (r‑TAR). Video report and results of a cohort study
Source: Chirurg. 2021 Aug 18;92(10):936–47. [Article in German] doi: 10.1007/s00104-021-01480-z (PMC8463520; doi:10.1007/s00104-021-01480-z)
Supplement: Supplementary file 2 [file 104_2021_1480_MOESM2_ESM.pdf]

Patientenaufkleber

Datum: _____/_____/_____

Operateur: _____________

r-TAR-Hernie

Transversus Abdominis Release

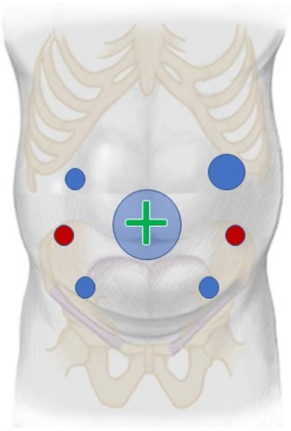

Rückenlage
Beide Arme angelagert
Patienten aufklappen

Intraoperatives Umdocken

daVinci Anatomie:
- Abdominal oben

daVinci Position:
- Patient rechtes

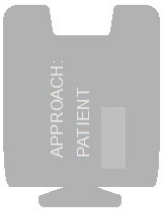

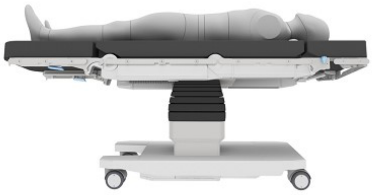

Perioperative Prozeduren/daVinci-Checkliste

(Achtung: Ersetzt nicht das WHO Team-Time-Out)

Ich habe den Aufklärungsbogen heute gesichtet und auf Inhalt und Bemerkungen hin kontrolliert: □ Ja □ Nein
Perioperative Antibiose: □ Ja □ Cefuroxim 1,5g (Gewichtsadaptiert) (Alternativ: □ Clindamycin 600mg)
Hybrid-Verfahren: □ Ja □ Nein Wenn ja, Ansage an OTA's machen.

Vor Andocken: **Für Double Docking vorbereiten.**

	Abstand der Trokar-Linie zur Linea alba mindestens 15cm:	□ Ja	□ Nein
	Es handelt sich nicht um ein Rezidiv:	□ Ja	□ Nein
	Anzahl der Voroperationen bzw. Adhäsionsvorgeschichte bekannt:	□ Ja	□ Nein
	Ropivacain-Infiltration der Portinzisionen vor dem Schnitt erfolgt:	□ Ja	□ Nein
	Laparoskopische Sichtkontrolle der Trokareinstischstellen:	□ Ja	□ Nein

**Konsole**:	Begin von links-lateral:	□ Ja	□ Nein
	Komplette Adhäsiolyse der vorderen Bauchdecke:	□ Ja	□ Nein
	Einstieg in die kontralaterale Rektusscheide an der Linea alba:	□ Ja	□ Nein
	Transversus abdominis release, top-down (gelber Pfeil) oder down-to-up (grüner Pfeil)		
	Präparation an Rippe, Zwerchfell und Xiphoid, komplette craniale Durchtrennung der posterioren Rektusscheide:	□ Ja	□ Nein
	Präparation im Retzius- und Bogros-Raum:	□ Ja	□ Nein
	Umdocken, analoge Präparation von rechts, Extraperitonealisierung der Ports	□ Ja	□ Nein
	Ausschluss von peritonealen Läsionen (ggf. Nähen):	□ Ja	□ Nein
	Mediane Naht der hinteren Rektusscheide (V-Loc 0):	□ Ja	□ Nein
	Mediane Naht der vorderen Rektusscheide mit Bruchsack (V-Loc 0):	□ Ja	□ Nein
	Netz-Messung, -Einrollung (mit Faden) und -Positionierung:	□ Ja	□ Nein
	Revision der Blutstillung und Arista Bestäubung:	□ Ja	□ Nein
	**Entlastung des Pneumopräperitoneums (3min) oder RR über 160mmHg**	□ Ja	□ Nein
	Einlage von 2 passiv-Drainagen (rechts und links):	□ Ja	□ Nein

**Abschluss**:	**Hybrid-Hautresektion:**	□ Ja	□ Nein
	Fasziennaht des 12mm Portzugangs mit Endoclose (fakultativ):	□ Ja	□ Nein
	OP-Daten in das Register eingetragen:	□ Ja	□ Nein
	Sichtkontrolle der Port-Entfernung:	□ Ja	□ Nein

Prästationäre Vorbereitung:
CT-Abdomen mit transversalen und sagittalen Schnitten (Morphologie und Verwachsungen)
Würzburg-Inzisionalhernienklassifikation

Nahtmaterial und Aushilfsmaterialien:
V-Loc 180 (grün) 0 USP GS-21 (30cm)
Vicryl 2-0 SH (20cm)
Vicryl 3-0 ohne Nadel für Netz-Einrollung
Lineal
Versatex Netz 30x30cm
Markierstift
2x passiv-Drainagen #18
Arista AH
Arista FlexTip XL-R Applicator

Lagerung:
Rückenlage
Keine Pink-Pad
Beide Arme ausgelagert

Geplante OP-Zeit: 4-6h

CIRS Meldung: □ Ja □ Nein

Instrumente:
30° Optik
Prograsp Forceps
Mega SutureCut Needle Driver
Hot Shears MCS
5x 8mm daVinci Ports
1x 12mm daVinci Ports

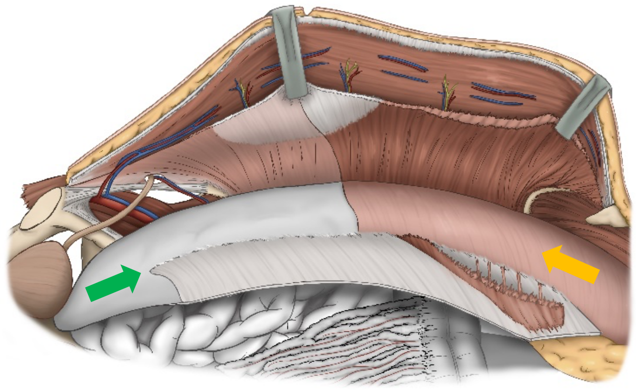

Dietz (Hrsg.) (2021) Offene Hernienchirurgie. Springer Verlag.

Hautnaht:
Ropivacain 0,5% 10ml
Caprosyn 4-0 USP mit P-24 Nadel
Glubran

Postoperative Verordnung:
Unbedingt PONV-Prophylaxe beachten
Thromboseprophylaxe
Mobilisation am Folgetag
Drainage 2-3 Tage belassen
Vollbelastung nach 3-14 Tagen
Informationsblatt aushändigen

DRG G09D, Zielverweildauer: **0** | 3 | 9 Tage

Geplante Entlassung: _____/_____/_____

Dietz at al (2007) J Plast Reconstr Aesthet Surg 60: 383-388.

Ocurrence	M = morphology	S = size (cm)	RF = risk factors (+ → +++)
p (*primary*)	m (*median*)	a x b	Obesity (BMI >25)
r (*recurrent*)	u (*umbilical*)		Male gender
	sp (*suprapubic*)		Nicotine abuse
	sc (*subcostal*)		Wound contamination
	t (*transversal*)		Age > 45 years
	l (*lumbal*)		Disease
	pm (*paramedian*)		2nd laparot. < 1 month
	nc (*other*)		Emergency procedure
			Postop. Complications
			…

v/i M… S…x… RF…

Debriefing: Was kann bei der nächsten OP besser gemacht werden?
